# Pediatric oncology patients prefer cocoa over oral nutritional supplements in a double-blinded feeding-trial

**DOI:** 10.3389/fnut.2026.1847634

**Published:** 2026-05-25

**Authors:** Mark Rassner, Hannes Buchner, Hans Bauer, Irene Schmid, Lisa Tenius

**Affiliations:** 1 Department of Pediatrics, Dr. von Hauner Children’s Hospital, University Hospital, LMU Munich, Munich, Germany; 2 Staburo GmbH, Munich, Germany

**Keywords:** chemotherapy, food acceptability, malnutrition, oral nutritional supplements, pediatric oncology, sensory evaluation, taste

## Abstract

**Background:**

Malnutrition is a common problem during the treatment of pediatric cancer. Therapy-induced taste alterations (dysgeusia) can further exacerbate this and may reduce the acceptance of oral nutritional supplements (ONS). There are no available data on the acceptability of commercially available ONS among children and adolescents in Germany.

**Methods:**

In a prospective, double-blind tasting trial, 36 pediatric oncology patients (6–20 years) evaluated commercially available ONS and a cocoa-based reference beverage. The primary outcome was overall liking (taste rating) on a 1–6 ordinal scale (1 = excellent; 6 = unacceptable). Additional assessments included ratings of appearance and temperature, perceived basic taste qualities, and free-text flavour descriptions. Using linear mixed models, beverage taste ratings were compared globally and pairwise (adjusted for multiple comparisons), and sensory characteristics associated with higher acceptance were explored.

**Results:**

Cocoa was rated best overall (mean taste rating 1.42 ± 0.7) and significantly outperformed all tested ONS in taste (Bonferroni-corrected *p* = 0.025 compared to next-best), appearance, and temperature. The best-rated supplements were predominantly sweet or sweet–sour (red fruits: 2.42 ± 1.3; summer fruit smoothie: 2.64 ± 1.5); the other products were rated rather poorly (all ≥3.47) and most were not accepted (4.0). For salty/savoury beverages, patients’ qualitative taste descriptions frequently diverged from manufacturer-declared flavours. Ratings of appearance and temperature, along with perceived sweetness, and in many cases sourness, emerged as the most robust predictors of overall taste evaluations. However, a model comparison indicated that these characteristics did not fully explain between-beverage differences (likelihood-ratio test *p* < 0.001), suggesting additional unmeasured determinants of acceptance.

**Conclusion:**

Among patients in pediatric oncology, receiving chemotherapy, cocoa was consistently preferred over commercially available high-energy ONS. While cocoa served as a highly accepted reference beverage, the findings highlight the importance of flavour characteristics for acceptance. Strategies focusing on flavour optimization of nutritionally complete supplements may help improve oral intake during therapy. In addition to fruit-based beverages, cocoa-based formulations – particularly when combined with energy enrichment approaches – may represent a practical and cost-efficient strategy to support oral nutritional intake when acceptance of standard ONS is limited.

## 1 Introduction

Approximately 2.250 children in Germany (1) and 400.000 children worldwide (2) are diagnosed with cancer annually, making it one of the leading causes of disease-related death in childhood.

In addition to the overall disease burden, intensive therapeutic regimens, including surgery, radiotherapy, and chemotherapy, exert a profound impact on physical well-being and health-related quality of life (3).

Specifically, malnutrition, defined as a state of deficiency, excess, or imbalance in an individual’s intake of energy and/or nutrients (4), is a critical concern in both adult and pediatric populations undergoing chemotherapy, with prevalence estimates indicating that 60–75% of all children are affected (5, 6). Although malnutrition may manifest as undernutrition (including wasting, stunting, underweight, and micronutrient deficiencies) or overnutrition (such as overweight and obesity) (4), prevalence estimates vary considerably. Longitudinal data suggest that overweight and obesity may increase during pediatric cancer treatment, and, in some cohorts, become more prevalent than undernutrition (7). Concomitantly, malnutrition, at any time point, has consistently been associated with adverse clinical outcomes, including increased infection rates, reduced tolerance to chemotherapy, treatment interruptions, and decreased overall survival in pediatric oncology patients (6, 8–10). Notably, both undernutrition and weight loss (8–10), as well as overnutrition, obesity (11–14) and even the cumulative duration of weight extremes (either high or low) (12) have been identified as risk factors for treatment-related complications and adverse clinical outcomes, including poorer survival. Alterations in lean tissue and fat mass have been suggested to influence the volume of distribution and metabolism of chemotherapeutic agents, thereby potentially modifying the clearance of hydrophilic and/or lipophilic drugs from systemic circulation (10, 15–18). Furthermore, malnutrition has also been shown to impair physical functioning and quality of life, thereby substantially affecting patients’ overall well-being during therapy (6).

Several factors contribute to malnutrition during chemotherapy. Nausea, mucositis, and diminished appetite are common adverse effects of treatment. However, despite often being under-recognized in clinical practice, alterations in taste appear to constitute the primary drivers of reduced interest in food and meal omission, at least among adult patients (19, 20). Taste receptor cells have high division rates, making them especially vulnerable to chemotoxicity (21–23), and consequently changes in taste and smell perception are frequently observed among pediatric cancer patients (21, 24–26). Altered taste perception has been linked to inadequate food or dietary intake in several pediatric studies (21, 25, 27–29), and such taste changes may indicate patients who are particularly vulnerable to food-related challenges and weight loss (27, 30, 31). Moreover, irrespective of nutritional outcomes, alterations in taste and smell perception are reported to be among the most frequent and distressing subjective adverse effects during treatment, significantly affecting children’s daily lives (25, 26, 32, 33). Remarkably, both decreases and increases in specific taste qualities have been documented (25), and different studies report heterogeneous effects on the perception of sour, bitter, sweet, or salty stimuli. However, the subjective hedonic experience of eating—often referred to as ‘liking of food’ or ‘food hedonics’—appears to be adversely affected in relation to taste alterations, irrespective of the direction of these changes (21, 25). Furthermore, although most studies with pediatric and adult patients report a preference for salty or savoury foods (4, 33), recent analyses indicate a reduced preference for sweet tastes (25), in contrast to earlier findings.

To mitigate malnutrition and prevent weight loss, a range of therapeutic strategies has been implemented, including oral nutritional supplements (ONS), appetite stimulants, enteral nutrition (EN), and parenteral nutrition (PN) (34–37). ONS, typically classified as Foods for Special Medical Purposes, are specially formulated oral products designed to prevent or treat malnutrition by augmenting habitual dietary intake with additional energy and essential nutrients. In principle, the beneficial effects of ONS consumption—as well as of appetite stimulants—on weight status, growth, and overall nutritional status have been demonstrated in several studies in pediatric oncology patients or children at risk for malnutrition (34, 36–39). For instance, in a randomized clinical study, Gokcebay et al. (38) reported that protein- and energy-dense ONS helped to prevent weight loss and improve anthropometric parameters. Similarly, Liang et al. showed that ONS improved nutritional status, reduced treatment-related complications, and lowered hospitalization costs for children with acute lymphoblastic leukemia (39). Such findings support the clinical use of ONS as a cornerstone strategy in the nutritional management of pediatric cancer patients. However, in most of these studies, only a single—albeit different—ONS preparation was provided, and the supplements were evaluated in highly heterogeneous patient populations (e.g., newly diagnosed versus in remission, differing treatment durations, etc.) (34, 38, 39). None of these studies assessed overall dietary intake beyond the ONS, and, importantly, they did not examine compliance with or frequency of ONS use (34). Given that taste is one of the most influential determinants of acceptance of (new) food products (19), a rigorous evaluation of ONS acceptability during chemotherapy is essential for the effective management of malnutrition. Nevertheless, such assessments remain scarce. To date, the acceptability of commercially available ONS has been systematically examined in only a single pediatric study conducted in Australia (40), in which five supplements were compared in a double-blind design involving 21 patients. Milk-based, commercially available drinks were preferred, especially when the brand was revealed.

In Germany, no data are currently available on which commercially available supplements are preferred or rejected by children and adolescents, nor the underlying reasons for these preferences. The present study was therefore designed as an exploratory investigation to evaluate the acceptability of a broad range of commercially available oral nutritional supplements in pediatric oncology patients, with overall liking as the primary outcome. Using a prospective, double-blind tasting design, we compared the ratings of eight supplements with those of a cocoa-based reference beverage, representing a commonly accepted option. We additionally investigated whether basic taste perceptions and sensory characteristics could account for differences in overall taste ratings across beverages. By identifying which products are most acceptable, and which sensory attributes predict acceptance, this work aims to inform practical strategies to support oral nutritional intake during chemotherapy.

## 2 Methods

Thirty-six patients between 6 and 20 years of age were recruited from the Department of Oncology at Dr. von Hauner Children’s Hospital, the Pediatric Clinic and Polyclinic of the Ludwig Maximilian University of Munich (LMU), where they were undergoing treatment for malignant neoplasms. Inclusion criteria required that patients had completed at least one course of chemotherapy prior to participation. This prospective study was conducted between February 2015 and October 2017 and was approved by the Ethics Committee of the Ludwig Maximilian University of Munich. Patients and their parents received both oral and written information through individualized information materials. Following parental consent, written assent was also obtained from the participating children. Exclusion criteria comprised age under 6 years, absence of informed consent, food allergies, and insufficient proficiency in German language.

Tasting sessions were scheduled at least 4 weeks after initiation of chemotherapy and prior to the start of subsequent treatment blocks, i.e., during clinically stable intervals between treatment cycles. Further, this scheduling was chosen to assess ONS acceptability under relatively stable conditions and to minimize the influence of acute treatment-related toxicities such as mucositis. The sessions were conducted on the ward by a dietitian. For tastings involving children aged 6 to <10 years, the presence of parents was permitted, provided that they did not exert any influence on the children’s responses. All tested products were commercially available ONS classified as Foods for Special Medical Purposes (FSMP), intended for the dietary management of pediatric patients with increased nutritional needs, and routinely used in pediatric oncology care. To represent the full range of available ONS, a total of eight different beverages based on six high-energy products and one normo-caloric product were selected under the supervision of the Department of Dietetics, including both sweet and salty or savoury options. All tested beverages are described in Table 1, including standardized beverage IDs, commercial product names, manufacturers, and nutritional composition. No specific manufacturers were preferentially selected. To ensure full reproducibility, a detailed ingredient list for all tested products is provided in Supplementary Table S1. Since there are few savoury oral nutritional supplements available, a normocaloric product was also included, to provide more variety. As one supplement was discontinued by the manufacturer during the course of the study, some patients received a beef/zucchini-based product, whereas others received a chicken/tomato/fennel-based product. At the time of manuscript preparation, all investigated products – except for the savoury formulation that was already discontinued during the study period – remain commercially available in unchanged formulations, indicating that the findings remain directly applicable to current clinical practice. In addition to tasting seven commercially available oral nutritional supplements, each patient was offered cocoa as a natural food item serving as a hypo-caloric reference beverage. All beverages were administered in opaque 10 mL cups, and patients were instructed to consume 10 mL of water between each sample. No packaging or product names were disclosed, and the order of sample presentation was randomized for each participant to minimize potential order effects and bias in taste evaluation, such as fatigue-related bias, thereby allowing the tasting to be conducted in a double-blind manner. After each beverage, patients were asked to complete a structured questionnaire consisting of five items. First, they were asked to identify the perceived taste quality (sweet, sour, salty, or bitter) and whether a specific flavour could be assigned. Subsequently, children were asked to rate appearance, temperature, and taste on a scale from 1 to 6, where 1 indicated *excellent*, 2 *good*, 3 *acceptable*, 4 *adequate*, 5 *poor*, and 6 *very poor*/*unacceptable*. The primary outcome was overall liking (taste rating) assessed on this scale. Although the rating scale represents an ordinal measure, it was treated as a continuous variable for the purpose of linear mixed model analysis, as is commonly done for Likert-type scales in comparable studies.

**Table 1:** Characteristics and nutritional values of oral nutritional supplements (ONS) included in study.

ID	Study label/flavour	Commercial product	Manufacturer used	Ingredients	Values for 100 mL					
					kcal	kJ	Prot.	Fat	Carb.	Fibers
B1	Cocoa	Custom preparation (cocoa drink)	Mondelez/Krüger Berchtesgadener Alpenmilch	12.5 g Cocoa + 100 mL milk 3.5%	114	479	4	4.4	15	1.7
B2	Smoothie red fruits	NutriniDrink Smoothie Red Fruits	Nutricia	Ready-to-drink	150	625	3.4	6.4	19.0	1.4
B3	smoothie summer fruits	NutriniDrink Smoothie Summer Fruits	Nutricia	Ready-to-drink	150	625	3.4	6.4	19.0	1.4
B4	ONS powder	EnergeaP Kids	MetaX Berchtesgadener Alpenmilch	12.5 g ONS powder +100 mL milk 3.5%	121	507	4.8	7	12	1.6
B5	ONS powder enriched with cocoa	Custom preparation (EnergeaP Kids + cocoa)	MetaX Mondelez/Krüger Berchtesgadener Alpenmilch	12.5 g ONS powder +12.5 g Cocoa +100 mL milk 3.5%	170	714	5.4	7.4	22	1.3
B6	Milk/banana	HiPP enteral formula milk/banana	HiPP	ready-to-drink	150	628	6.3	5.3	17.9	2.5
B7	Beef/zucchini	HiPP enteral formula beef/zucchini	HiPP	ready-to-drink	100	419	3.7	3.7	12.2	1.5
B8	Pumpkin/carrot	HiPP enteral formula pumpkin/carrot	HiPP	ready-to-drink	150	628	6.3	5.3	17.9	2.5
B9	Chicken/tomato/fennel	HiPP enteral formula chicken/tomato/fennel	HiPP	ready-to-drink	150	628	6.3	5.3	17.9	2.5

Prot., proteins; Carb., carbohydrates; Ctrl., control.

Patient-related data were pseudonymized through the assignment of study using identification numbers and were processed exclusively in their pseudonymized form. Compliance with physician–patient confidentiality was rigorously maintained throughout the study.

Quantitative variables are presented as indicated using descriptive statistics (mean, standard deviation, standard error of the mean, minimum, and maximum). For categorical variables and free-text variables, absolute and relative frequencies of categories or participant responses are reported. Comparisons between beverages and their qualities were based on linear mixed models (LMM) with a random intercept, which accounts for inter-individual variability between patients. A likelihood-ratio test comparing a random intercept model with beverage as a fixed effect against a random intercept-only model was performed to assess overall differences between beverage taste ratings. Upon detection of a significant overall effect, pairwise contrasts between beverages (based on t-statistics with Kenward-Roger degrees-of-freedom correction) were performed to identify specific differences (Table 2). To control for multiple comparisons, *p*-values were adjusted using the Bonferroni correction. To identify beverage characteristics that may influence patient acceptance (Table 3), in a first step, model selection was performed to obtain the best-fitting set of predictors. Candidate predictors comprised ratings of appearance and temperature, as well as assessments of whether the beverage was perceived as sweet, sour, salty, bitter, or tasteless, for a total of 2^7^ = 128 candidate random-intercept LMMs. The category ‘other taste’ was excluded due to its low frequency and heterogeneity. Model selection was based on Akaike’s Information Criterion (AIC). The effects of predictors included in this model were interpreted with respect to their association with taste ratings. Subsequently, a likelihood-ratio test between the selected model with versus without beverage as an additional predictor was applied to assess whether differences in taste ratings between beverages could be fully explained by the selected predictors alone. A significant result would indicate that additional, unmeasured beverage characteristics beyond appearance, temperature, or basic taste perception likely contributed to differences in taste ratings.

**Table 2:** Evaluation of oral nutritional supplements

ID	Taste perception				Appearance		Rating of temp.		Rating of taste		
	Sweet	Sour	Salty	Bitter	No.	Mean (SD)	No.	Mean (SD)	No.	Mean (SD)	Group*
B1	97% (35/36)	0% (0/36)	0% (0/36)	6% (2/36)	36	1.4 (0.6)	36	1.6 (0.8)	36	1.42 (0.7)	1
B2	89% (31/35)	3% (1/35)	3% (1/35)	9% (3/35)	36	2.5 (1.5)	35	2.1 (1.3)	36	2.42 (1.3)	2
B3	53% (19/36)	67% (24/36)	8% (3/36)	14% (5/36)	36	2.6 (1.2)	36	2.0 (1.1)	36	2.64 (1.5)	2/3
B4	32% (11/34)	3% (1/34)	24% (8/34)	38% (13/34)	36	2.7 (1.7)	35	2.4 (1.4)	36	4.00 (1.7)	4/5
B5	81% (21/26)	0% (0/26)	8% (2/26)	31% (8/26)	25	2.1 (1.4)	26	1.8 (1.1)	26	3.47 (2.0)	3/4
B6	64% (23/36)	6% (2/36)	14% (5/36)	17% (6/36)	35	3.4 (1.6)	35	2.5 (1.5)	35	4.19 (1.6)	4/5
B7	50% (5/10)	0% (0/10)	50% (5/10)	10% (1/10)	10	3.7 (1.2)	10	3.3 (1.7)	10	4.48 (1.2)	4/5
B8	27% (9/33)	3% (1/33)	30% (10/33)	33% (11/33)	35	3.5 (1.5)	34	2.8 (1.5)	34	4.71 (1.2)	5
B9	28% (7/25)	16% (4/25)	32% (8/25)	28% (7/25)	25	3.6 (1.7)	24	2.5 (1.5)	25	4.77 (1.5)	5

*Non-overlapping numbers between any two table rows indicate statistically significant pairwise difference in taste rating at Bonferroni-corrected 5% leve. Temp., temperature; No., number; SD, standard deviation.

**Table 3:** Predictive models for ONS taste rating (TOP 10).

Included predictors in model	AIC
Appearance, temperature, sweet?, sour?	878.86
Appearance, temperature, sweet?, sour?, bitter?	879.86
Appearance, temperature, sweet?, sour?, tasteless?	880.20
Appearance, temperature, sweet?, sour?, salty?	880.27
Appearance, temperature, sweet?, sour?, bitter?, tasteless?	880.65
Appearance, temperature, sweet?, sour?, salty?, bitter?, tasteless?	880.73
Appearance, temperature, sweet?, sour?, salty?, bitter?	880.95
Appearance, temperature, sweet?, sour?, salty?, tasteless?	881.15
Appearance, temperature, sweet?, salty?, bitter?, tasteless?	883.21
Appearance, temperature, sweet?, bitter?, tasteless?	884.64

## 3 Results

### 3.1 Patient characteristics

A total of 36 pediatric patients treated for malignant neoplasms were included in the present study (Table 4). Mean age at inclusion was 12.44 years (±4.12 years). There was an even number of male (52.8%) and female (47.2%) patients. Mean weight at evaluation was 44.86 kg (±19.31 kg) with a mean height of 154 cm (±21.34 cm), equivalent to a mean target weight for height of 95 ± 18%. Clinically relevant mucositis was largely absent at the time of testing; only a single patient (2.8%) showed signs of mild mucositis during the assessment. Among the 36 patients, a total of 20 different underlying conditions were represented. Hematological malignancies constituted the largest subgroup, followed by central nervous system tumors and embryonal tumors (including nephroblastoma, hepatoblastoma, and rhabdomyosarcoma). On average, tasting sessions took place approximately 2.8 months after diagnosis, following completion of at least one chemotherapy cycle.

**Table 4:** Patient demographics and clinical characteristics.

Variable	Total, *N* = 36 (min-max)
Age (years)	
Mean ± SD	12.44 ± 4.12 (6–20)
Gender	
Male	19 (52.8%)
Female	17 (47.2%)
Weight (kg)	
Mean ± SD	44.86 ± 19.31 (16.4–87.0)
Height (cm)	
Mean ± SD	154 ± 21.34 (113–186)
Target weight for height (kg/kg)	
Mean ± SD	0.95 ± 0.18 (0.70–1.41)
Mucositis	yes: 1 (2.8%), no: 35 (97.2%)
Entities	
Leukemia, MDS	12 (33.3%)
Lymphoma	6 (16.7%)
CNS tumors	6 (16.7%)
Embryonal tumors (excl. CNS)	6 (16.7%)
Bone and soft-tissue sarcoma	4 (11.1%)
Other	2 (5.6%)
Time after diagnosis (mo.)	
Mean ± SD	2.83 ± 2.20 (1–9)

SD, standard deviation; MDS, myelodysplastic syndrome; CNS, central nervous system; mo., months.

### 3.2 Perception and ratings of oral nutritional supplements

Participants were asked to evaluate a total of seven individual commercially available products, as well as one additional modified beverage variant (see below). In addition, cocoa was offered as hypocaloric reference beverage control, and nutritional values of all beverages are given in Table 1. To capture the full spectrum of flavour profiles found in oral nutritional supplements, foods representing sweet, sour, and salty tastes were included. While most of the included supplements were acquired as pre-prepared drink, one beverage (ONS powder) was prepared fresh by mixing high-caloric powder in milk. Moreover, to explore whether simple flavour modification could improve acceptability, an additional ONS preparation was tested with the addition of cocoa to the aforementioned ONS powder formula (ONS powder 12.5 g/100 milk + cocoa 12.5 g/100 milk). This modification with cocoa was applied only to the powder-based product, as comparable flavour combinations were not feasible for all tested formulations, particularly savoury products. Unfortunately, one supplement (beef/zucchini) was discontinued by the supplier, so that it was offered to only a part of the cohort (10/36), and was subsequently replaced by another, similarly salty flavour (chicken/tomato/fennel) for the remainder of the study. Food tastings were conducted in a double-blind manner, with small samples served in opaque 10 mL cups. Between individual samples, patients were instructed to consume 10 mL of water (see methods).

After tasting each oral nutritional supplement, children were asked to rate (i) taste flavour—with inclusion of the four main taste perceptions, (ii) appearance, (iii) Beverage temperature, and finally iv. overall liking of the food supplement (Table 2). Additionally, they were asked to describe the overall taste flavour in their own words (Table 5). In order to facilitate evaluation for patients, ranking was conducted on an ordinal scale on the basis of school grades, ranging from 1 (excellent) to 6 (unacceptable).

**Table 5:** Qualitative descriptions of taste (patients’ own words).

ID	Option (no. of ratings/drink)	Taste perception (5 most prevalent)
B1	Cocoa (35)	Cocoa/kaba/chocolate 97.1% Very milky cocoa 2.9%
B2	Smoothie red fruits (33)	Strawberry 33.3% Banana 15.2%, milk 15.2% Cocoa/chocolate 12.1% Vanilla 9.1%
B3	Smoothie summer fruit (31)	(Multi)fruit 16.1%, orange 16.1% Lemon 12.9%, apricot 12.9% Banana 9.7%, passion fruit 9.7%
B4	ONS powder in milk + cocoa (24)	Cocoa/chocolate 66.7% Nut 12.5%, paper/cardboard 12.5% Strange (incl. “aftertaste”) 8.3% Disgusting (incl. “rabbit food”) 8.3%
B5	ONS powder in milk (31)	Milk 45.2% Paper/cardboard 12.9%, Oat/soy/almond/flour milk 12.9% Nut 6.5%, nothing 6.5%
B6	Milk/banana (24)	Fruits (incl. Apple, fig) 29.2% Banana 20.8% Candy/gum 12.5%, Milk 8.3%, caramel 8.3%
B7	Beef/zucchini (7)	Inexplicable/any ONT 28.6% Oat flakes 14.3%, cocoa 14.3% Banana 14.3%, mushrooms 14.3%, sweet & salty 14.3%
B8	Pumpkin/carrot (24)	Oat 12.5%, milky 12.5% Flour/wheat 12.5% Mango 8.3%, nothing 8.3%
B9	Chicken/tomato/fennel (15)	Pumpkin 20%, caramel 20%, Milk 13.3%, banana/passion fruit 13.3%, Cardboard/wood 13.3%

The total quota of subjects reporting each individual flavour perception (sweet, sour, salty, bitter) was calculated, allowing for multiple selections for each ONS, thereby allowing for mixed flavour perceptions (Table 2). Reflecting inclusion of several sweet food supplements, primary taste flavour perception was sweet. While two food supplements (cocoa, red fruits) were perceived primarily with a single flavour (sweet), perception of all other food supplements was of mixed nature. For one beverage (the ONS powder), the addition of cocoa reduced the perception of salty and bitter tastes, with a concurrent shift toward sweetness; overall, salty and sweet taste perceptions were largely mutually exclusive across all tastings (data not shown). Both for appearance and for temperature, there was a wide range in ratings. Overall, oral nutritional supplements that received more favourable ratings for their appearance tended to perform similarly well with respect to the perceived serving temperature.

With the exception of the control product, taste ratings were consistently lower than those for appearance or temperature. There were clear differences in mean beverage taste ratings (likelihood ratio *p* < 0.001). The overall mean across all food supplements was only 3.84, in contrast to the reference beverage (cocoa), which was rated best (1.42 ± 0.7) and significantly outperformed all included oral nutritional supplements. Descriptively, cocoa also outperformed all other beverages, with respect to optics and perceived temperature. Two food supplements that were perceived predominantly sweet (red fruit) or sweet and sour (smoothie summer fruit), respectively, achieved the second-best taste rating (2.42 ± 1.3, 2.64 ± 1.5). All other food supplements were rated average (3.47) or below (≥4.00).

When children were asked to describe taste of the beverage in their own words (Table 5), we observed a discrepancy between primarily sweet versus salty or savoury food supplements: Whereas the described or predominantly perceived flavours of sweet oral nutritional supplements largely matched the flavours stated by the manufacturer (cocoa, red fruit smoothie, summer fruit smoothie), this was not the case for salty or savoury beverages. In these cases, the manufacturer-declared flavour was not perceived by patients and instead was perceived as a completely different flavour.

### 3.3 Impact of beverage characteristics on taste ratings

Perceived taste qualities (e.g., sweet, sour, salty, bitter) reflect sensory perception and are conceptually distinct from overall liking (taste rating). To assess the extent to which taste ratings could be attributed to specific beverage characteristics, we employed an exhaustive model-selection approach (Table 3). Seven candidate predictors were considered (rating of appearance, temperature rating, and whether the drink was perceived as sweet, sour, salty, bitter, or tasteless). To identify the best combination of these predictors, we fitted every possible model that can be formed from these seven variables. Rating of appearance and temperature, and presence/absence of sweet taste were included in the ten strongest prediction models (Table 3), consistent with beverage taste ratings, in which sweet and/or sour drinks (cocoa, red fruit smoothie, smoothie summer fruits) were rated better (Table 2). Accordingly, presence of sour taste was also associated with better prediction of total taste rating (8/10), whereas (absence of) bitter, salty or tastelessness, was more infrequently included in the strongest prediction models.

We subsequently identified the best prediction model based solely on beverage characteristics (temperature, appearance, taste flavour), i.e., the model with the lowest Akaike Information Criterion (AIC), and compared its predictive power to that of the same model but with beverage added as a predictor. A likelihood-ratio-test (Table 6) showed that differences between beverages in taste ratings cannot be explained from the selected food characteristics alone (*p* < 0.001), meaning that there are other, but unknown characteristics, relevant for the taste ratings.

**Table 6:** Test of explanatory power of selected characteristics.

Model	AIC	Chi^2^	dfChi^2^	pChi^2^
Selected beverage characteristics	878.9			
+ Beverage	829.8	65.06	8	<0.001

AIC, Akaike Information Criterion; Chi², chi-square test statistic for the likelihood ratio test. dfChi², degrees of freedom of the chi-square test statistic. pChi², p-value of the chi-square test statistic.

## 4 Discussion

Malnutrition is a common and clinically relevant problem during chemotherapy. In addition to therapy-related nausea and mucositis, high rates of altered taste and smell contribute to impeded nutritional intake. Although oral nutritional supplements are widely used to counteract malnutrition, their acceptability during chemotherapy has been studied only to a minimal extent, and there is no data from Germany regarding which commercially available supplements are preferred or rejected by children and adolescents.

In our study, 36 pediatric oncology patients were asked to taste and evaluate seven commercially available oral nutritional supplement products, plus a cocoa reference beverage, in a double-blind, prospective manner. We selected a diverse patient cohort representative for disease incidence rates and reflecting distinct chemotherapy regimen backgrounds. While all patients had finished at least one round of chemotherapy, mucositis was present in only one of the included patients at the time of testing. To account for the additional burden of tasting sessions in a vulnerable patient population undergoing chemotherapy, tastings were conducted during intervals between treatment blocks, when patients were in a comparatively stable clinical condition, which also helped to minimize potential fatigue-related effects. Overall, cocoa was clearly the best rated beverage. It was rated best both for appearance, temperature and taste (Table 2), albeit a mutual influence of one feature’s rating on another’s cannot be ruled out (i.e., a positive taste perception may influence rating of appearance or vice versa, respectively). All other drinks were rated significantly lower than cocoa (*p* ≤ 0.025). Two additional sweet or predominantly sweet beverages (smoothie red fruit and smoothie summer fruits) received the second-highest ratings. With the exception of one mixed product (milk plus ONS powder and cocoa), all other supplement options were not accepted by the patients (overall rating ≥4.0). Notably, supplementation with cocoa shifted the main taste perception of the commercially available ONS powder from bitter to sweet. This finding represents an exploratory example of flavour modification. Nevertheless, despite improved rating (3.47 with vs. 4.00 without cocoa), the modified, sweeter product was still rated significantly worse than the highest-rated alternatives, including cocoa itself. Accordingly, although sweet and sour taste as well as a pleasant perception of appearance and temperature were statistically associated with higher overall acceptance, evaluation of an exhaustive selection model (Table 3) indicated that these factors alone were insufficient to fully explain beverage taste ratings. Consequently, additional factors contribute to supplement taste acceptance. The observation that, in contrast to the more highly rated alternatives, the less favorably evaluated beverage options were associated with a wide range of taste associations that diverged from the manufacturer-declared flavour, suggests that familiarity with the product may contribute to its evaluation. This interpretation is consistent with findings of Cohen et al. (40), who reported higher ratings for commercially available oral nutritional supplements when the brand was disclosed. Additionally, even though all patients were able to identify cocoa correctly, the observed variability in ONS preference may, at least in part, be explained by treatment-related alterations in taste and smell. Studies exploring taste perception have shown that children undergoing chemotherapy frequently experience sensory changes with altered food preferences and appetite, often starting early during treatment. It should be noted that the assessment of basic taste qualities (e.g., sweet, sour, salty, bitter) reflects patients’ perception of sensory attributes rather than their hedonic evaluation. While certain perceived taste qualities, particularly sweetness and sourness, were associated with higher overall liking in our study, these findings do not imply a direct causal relationship between taste perception and preference. Rather, perception and liking represent distinct but interrelated constructs, and their interaction may be influenced by additional factors such as familiarity or individual taste preferences. For example, van den Brink et al. reported that pediatric patients commonly perceive foods differently, with shifts in taste intensity and a tendency to prefer savory over sweet items (25). Such chemosensory changes can substantially influence food acceptance and may therefore contribute to the acceptance or rejection of specific ONS formulations. These findings highlight the importance of considering individual sensory preferences when selecting or prescribing ONS in pediatric oncology care.

## 5 Limitations

It should be noted that the cocoa-based beverage cannot be considered a neutral comparator, as such drinks are generally well accepted among children. Rather than serving as a placebo, cocoa was intentionally included as commonly preferred reference product, and while this may have introduced a hedonic bias influencing comparative ratings, the aim was to provide a clinically meaningful benchmark for comparison. At the same time, the consistent identification and high rating of cocoa across patients may indicate relatively preserved taste perception during chemotherapy, in contrast to the more heterogeneous perception of ONS.

A limitation is that one of the tested products was replaced during the study period (beef/zucchini replaced by chicken/tomato/fennel), resulting in two different but similar savory formulations being evaluated in subsets of patients. Consequently, not all participants assessed all products, leading to a partially unbalanced study design. However, both products were comparable in terms of their savory taste profile and intended use, and were therefore considered functionally equivalent for the purposes of this study. Nevertheless, this aspect may have limited the direct comparability between all tested products.

With the exception of one patient, patients with mucositis were not assessed in our study, since tastings were conducted during intervals between chemotherapy blocks. Given that mucositis is known to substantially affect taste perception, our findings may not be fully generalizable to patients experiencing active mucosal toxicity. Future studies may specifically address supplement acceptability in this clinically relevant group.

A further limitation relates to the potential for carry-over effects between samples. While patients were instructed to consume 10 mL of water between tastings, no formal washout protocol was implemented. This approach reflected a pragmatic compromise in a pediatric oncology setting. However, particularly in younger children, it may not have been sufficient to fully neutralize strong or persistent flavours with potential carry-over bias in subsequent ratings. More rigorous washout procedures were not considered feasible due to the clinical condition and age of the participants, and to minimize additional burden during the tasting sessions.

## 6 Conclusion

In conclusion, data from this study suggest that overall liking of nutritional beverages in pediatric oncology patients is strongly influenced by sensory characteristics, particularly sweetness and familiarity. Cocoa can be offered as a low-cost option to support oral nutritional intake. Moreover, it may serve as a practical benchmark for acceptable flavour profiles, and strategies focusing on flavour optimization appear promising to improve intake of high-caloric nutritional supplements.

For example, combining nutritionally complete supplements with familiar taste components – such as cocoa – may enhance acceptability. In this context, enrichment of 100 mL milk (65 kcal) with just 12.5 g cocoa (49 kcal) and 12.5 g of the high-caloric ONS powder (56 kcal), respectively, results in a caloric density even higher than that of high-energy supplements (approx. 170 kcal/100 mL vs. 150 kcal/100 mL). However, such preparations primarily increase caloric intake and do not replace the full nutritional composition of commercially available ONS. In addition, offering other sweet and familiar beverages—potentially drinks similar to the two fruit-based smoothies included in this study—may further enhance acceptance of nutritional supplements. Similarly, consumption of these supplements should be avoided immediately prior to chemotherapy associated with gastrointestinal toxicity to prevent the development of conditioned food aversions (21, 41), whereby the beverage may become negatively associated with treatment-related gastrointestinal side-effects. Conversely, offering different supplements throughout therapy may help counteract taste fatigue. Finally, to clarify the complex relationship between altered taste perception and potential changes in food preferences, comprehensive studies including both patient and control groups (healthy individuals or patients assessed prior to the initiation of chemotherapy) are currently lacking. Such studies could help to better understand how exactly taste perception—or changes in taste perception—influence the acceptance of different nutritional supplements.

## Data Availability

The original contributions presented in the study are included in the article/Supplementary material, further inquiries can be directed to the corresponding authors.
