# Supplementary material for: Pediatric oncology patients prefer cocoa over oral nutritional supplements in a double-blinded feeding-trial
Source: Front Nutr. 2026 May 25;13:1847634. doi: 10.3389/fnut.2026.1847634 (PMC13243086; doi:10.3389/fnut.2026.1847634)
Supplement: Supplementary file 1 [file Data_Sheet_1.pdf]

SUPPLEMENTARY TABLE S1. Detailed ingredient composition of all products included in the study

ID	Product	Ingredients
**B1**	Cocoa	Sugar, heavily defatted cocoa powder (18%), dextrose (15%), emulsifier (lecithins), salt, flavouring.
**B2**	NutriniDrink Smoothie Rote Früchte[1] (Smoothie red fruits)	Water, fruit purée and fruit juice concentrate (15%) (strawberry purée (3.6%), raspberry purée (3.6%), apple purée (3.6%), pear purée (3.6%), lemon juice from concentrate (0.2%)), sugar, maltodextrin, vegetable oils (rapeseed oil, sunflower oil), cow's MILK protein, carrot juice concentrate (1.5%), dietary fibers (SOY polysaccharides, inulin, oligofructose, resistant starch, gum arabic, cellulose), acidity regulators (citric acid, phosphoric acid), flavourings (strawberry, raspberry), calcium lactate, stabilizer (pectin), emulsifier (mono- and diglycerides of fatty acids), potassium citrate, disodium phosphate, magnesium citrate, sodium citrate, dicalcium phosphate, choline chloride, carotenoids (contains SOY; beta-carotene, lutein, lycopene oleoresin from tomatoes), L-ascorbic acid, sodium L-ascorbate, taurine, ferric diphosphate, zinc sulphate, L-carnitine, sodium selenite, cholecalciferol, retinyl acetate, calcium D-pantothenate, D-biotin, nicotinamide, DL-alpha-tocopherol acetate, manganese sulphate, thiamine hydrochloride, copper sulphate, riboflavin, pyridoxine hydrochloride, potassium iodide, sodium fluoride, cyanocobalamin, phytomenadione, sodium molybdate, pteroylmonoglutamic acid, chromium(III) chloride.
**B3**	NutriniDrink Smoothie Sommerfrüchte[1] (Smoothie summer fruits)	Water, fruit purée and fruit juice concentrate (15%) (apricot purée (3.5%), banana purée (3.5%), apple purée (3.5%), pear purée (3.5%), lemon juice from concentrate (1.0%)), sugar, vegetable oils (rapeseed oil, sunflower oil), maltodextrin, cow's MILK protein, carrot juice concentrate (0.9%), stabilizer (pectin), dietary fibers (SOY polysaccharides, inulin, oligofructose, resistant starch, gum arabic, cellulose), acidity regulators (citric acid, phosphoric acid), emulsifier (mono- and diglycerides of fatty acids), flavouring, sodium citrate, calcium lactate, dicalcium phosphate, starch, potassium citrate, carotenoids (contains SOY; beta-carotene, lutein, lycopene oleoresin from tomatoes), choline chloride, magnesium citrate, L-ascorbic acid, sodium L-ascorbate, taurine, ferric diphosphate, zinc sulphate, L-carnitine, cholecalciferol, retinyl acetate, sodium selenite, nicotinamide, biotin, calcium D-pantothenate, copper sulphate, thiamine hydrochloride, manganese sulphate, riboflavin, pyridoxine hydrochloride, sodium fluoride, phytomenadione, pteroylmonoglutamic acid, sodium molybdate, chromium(III) chloride.
**B4**	EnergeaP Kids[1]	Maltodextrin, vegetable oils (palm oil, rapeseed oil, sunflower oil), milk protein, dextrin, skimmed milk powder, Vitamin B2, Vitamin B1, magnesium carbonate, emulsifiers: sunflower lecithin & E 472c, potassium carbonate, calcium phosphate, iron diphosphate, sodium carbonate, sodium chloride, Vitamin C, zinc sulphate, Vitamin E, sodium fluoride, Vitamin D, niacin, manganese sulphate, pantothenic acid, copper sulphate, Vitamin B6, Vitamin A, folic acid, chromium(III) chloride, sodium selenite, potassium iodide, sodium molybdate, Vitamin K, biotin, Vitamin B12.
**B6**	Sondennahrung mit Milch und Banane[1] (enteral formula with milk and banana)	Partially skimmed milk 48%, water, maltodextrin, banana juice from banana juice concentrate 6%, vegetable oils (rapeseed oil, sunflower oil), milk protein (lactose-reduced), soy protein, oat fibre, inulin, potassium hydroxide, potassium citrate, sodium citrate, magnesium citrate, emulsifier: lecithins (soy); emulsifier: citric acid esters of mono- and diglycerides of fatty acids; sodium chloride, acidity regulator: tricalcium phosphate; Vitamin C, iron citrate, niacin, zinc sulphate, Vitamin B1, manganese sulphate, Vitamin E, copper sulphate, pantothenic acid, Vitamin B6, Vitamin A, Vitamin B2, chromium chloride, folic acid, potassium iodate, Vitamin K, sodium selenate, biotin, sodium molybdate, Vitamin D, Vitamin B12.
**B7**	Sondennahrung mit Rind und Zucchini-Gemüse[1] (enteral formula with beef and zucchini)	Partially skimmed milk 48%, water, maltodextrin, zucchini 8%, parsnips 4%, vegetable oils (rapeseed oil, sunflower oil), beef 2.0%, soy protein, milk protein (lactose-reduced), oat fibers, potassium hydroxide, inulin, sodium citrate, emulsifier lecithins (soy), potassium citrate, magnesium citrate, Vitamin C, sodium chloride, acidity regulator tricalcium phosphate, iron citrate, niacin, zinc sulphate, Vitamin B1, Vitamin E, Vitamin B6, copper sulphate, manganese sulphate, pantothenic acid, Vitamin B2, Vitamin A, chromium chloride, folic acid, Vitamin K, potassium iodate, sodium selenate, sodium molybdate, biotin, Vitamin D, Vitamin B12.
**B8**	Sondennahrung mit Kürbis und Karotte[1] (enteral formula with pumkin and carrot)	Partially skimmed milk 48%, water, maltodextrin, vegetable oils (rapeseed oil, sunflower oil), pumpkin juice from pumpkin juice concentrate 4%, milk protein (lactose-reduced), carrots 3.0%, soy protein, oat fibre, inulin, potassium hydroxide, potassium citrate, sodium citrate, magnesium citrate, emulsifier: lecithins (soy); emulsifier: citric acid esters of mono- and diglycerides of fatty acids; sodium chloride, Vitamin C, acidity regulator: tricalcium phosphate; iron citrate, niacin, zinc sulphate, Vitamin B1, manganese sulphate, Vitamin E, copper sulphate, pantothenic acid, Vitamin B6, Vitamin A, Vitamin B2, chromium chloride, folic acid, potassium iodate, Vitamin K, sodium selenate, biotin, sodium molybdate, Vitamin D, Vitamin B12.
**B9**	Sondennahrung mit Huhn, Tomate und Fenchelgemüse[1] (enteral formula with chicken, tomato and fennel)	Partially skimmed milk 48%, maltodextrin, water, tomatoes 6%, pumpkin juice from pumpkin juice concentrate 4%, vegetable oils (rapeseed oil, sunflower oil), milk protein (lactose-reduced), soy protein, fennel 2.0%, chicken meat 2.0%, oat fibers, inulin, potassium hydroxide, potassium citrate, sodium citrate, magnesium citrate, emulsifier: citric acid esters of mono- and diglycerides of fatty acids, emulsifier: lecithins (soy), sodium chloride, Vitamin C, acidity regulator: tricalcium phosphate, iron citrate, niacin, zinc sulphate, Vitamin B1, manganese sulphate, Vitamin E, copper sulphate, pantothenic acid, Vitamin B6, Vitamin A, Vitamin B2, chromium chloride, folic acid, potassium iodate, Vitamin K, sodium selenate, biotin, sodium molybdate, Vitamin D, Vitamin B12.

[1] food for special medical purpose (FSMP) – complete formula
